# Norwegian population norms for the EQ-5D-5L: results from a general population survey

**DOI:** 10.1007/s11136-021-02938-7

**Published:** 2021-07-16

**Authors:** Andrew Malcolm Garratt, Tonya Moen Hansen, Liv Ariane Augestad, Kim Rand, Knut Stavem

**Affiliations:** 1 Division for Health Services, Norwegian Institute of Public Health, Oslo, Norway; 2 Department of Health Management and Health Economics, Medical Faculty, University of Oslo, Oslo, Norway; 3 Health Services Research Unit, Akershus University Hospital, Lørenskog, Norway; 4 Institute of Clinical Medicine, University of Oslo, Oslo, Norway; 5 Department of Pulmonary Medicine, Medical Division, Akershus University Hospital, Lørenskog, Norway

**Keywords:** Quality of life, EQ-5D, Population norms, General population, Postal survey

## Abstract

**Purpose:**

To provide the first Norwegian EQ-5D-5L and EQ VAS population norms for the adult general population.

**Methods:**

Postal survey of a random sample of 12,790 Norwegians identified through the National Registry of the Norwegian Tax Administration. Norms, weighted for Norwegian general population characteristics, are shown for the five EQ-5D-5L dimensions, EQ-5D index, and EQ VAS scores for seven age categories, females, males, and education level.

**Results:**

There were 3200 (25.9%) respondents to 12,263 correctly addressed questionnaires. The EQ-5D-5L dimensions, EQ VAS, and background questions were completed by 3120 (24.6%) respondents. The mean age (SD) was 50.9 (21.7) and range was 18–97 years. The youngest age group of 18–29 years and oldest of 80 years and over had the highest (*n* = 691) and lowest (*n* = 239) number of respondents, respectively. Compared to the general population, the respondents comprised a greater number of females, younger and older ages, and had a higher education level. 32% of respondents reported no health problems on the EQ-5D-5L. From the youngest to oldest age groups, there was a general decline in health as assessed by the EQ-5D-5L. The exception was for anxiety/depression, where the youngest age groups had the poorest health. Apart from self-care, women reported poorer health than men, as assessed by the EQ-5D-5L; EQ VAS scores were similar for men and women. Higher levels of health (EQ-5D index, EQ VAS scores) were found with increasing levels of education.

**Conclusion:**

The population norms will improve interpretation of EQ-5D-5L and EQ VAS scores in Norwegian applications including clinical practice, clinical and health services research, and national quality registers where EQ-5D-5L is the most widely used patient-reported instrument.

**Supplementary Information:**

The online version contains supplementary material available at 10.1007/s11136-021-02938-7.

## Introduction

The EuroQol EQ-5D is by far the most widely used patient-reported health outcome measure (PROM) suitable for use in economic evaluation including cost per quality adjusted life year (QALY) calculations [1, 2]. The instrument is available in over 150 languages [3], and national value sets and population norms for purposes of scoring and interpretation exist for over 20 countries [4, 5]. It is brief, widely tested, and includes five important aspects of health or dimensions (mobility, self-care, usual activities, pain/discomfort, and anxiety/depression). The original version of the instrument, now referred to as the EQ-5D-3L, assessed these aspects of health with three levels corresponding to no, moderate, and extreme problems [4]. The more recent EQ-5D-5L, used in this study, has five levels, corresponding to no, slight, moderate, severe, and extreme problems.

The EQ-5D is considered highly acceptable to most patient groups and feasible for application where a short-form general measure of health is required. The instrument has had widespread application in research including clinical trials, population health surveys, and as a health care quality indicator. The latter includes the National Health Service for England and Wales Patient-Reported Outcomes Measures (PROMs) program [4] and Norwegian and Swedish National Quality Registries (NQR) where it is the most widely used PROM [6, 7]. The Norwegian Medicines Agency recommends the use of EQ-5D in all technology assessments [8]. In the absence of a Norwegian value set and scoring algorithm, the Agency recommends the EQ-5D-3L algorithm for the UK [9] together with a mapping algorithm [10].

Population norms or reference scores have been widely used to aid the interpretation of PROMs, including short-form generic instruments available over the last three decades, such as the EQ-5D and SF-36 [11, 12]. Generic PROMs have relevance across populations irrespective of underlying health problems, and norms are usually based on representative samples of the general population. Population norms provide a benchmark or reference to interpret individual or group scores, often for specific health problems [11–13]. Norwegian population norms for the EQ-5D-3L recently became available and include data for the EQ-5D index, EQ VAS, and dimension scores for categories of age, sex, and education level [11].

National population norms are increasingly available for the EQ-5D-5L and to date include 14 countries: Bulgaria [14], China [15], Germany [16], Hong Kong [17], Italy [18], Indonesia [19], Ireland [20], Japan [21], Poland [22], Slovenia [23], Spain [24], Trinidad and Tobago [25], the USA [2], and Vietnam [26]. Data were collected by computer-assisted interview, face-to-face interview, online, and self-completed (pen and paper). The collection of population norms was the main objective of four studies, while the remainder had other objectives including valuation and national health surveys, which also included the EQ-5D-5L questionnaire. Samples size ranged from approximately 1000 [14], the level required for EQ-5D-5L valuation studies, to over 20,000 in the Spanish National Health Survey [24]. Most studies reported norms for the EQ-5D-5L dimensions, index, and EQ VAS scores across categories of age and sex, with other socioeconomic variables including education level, being less widely reported [2, 14–26].

This is the first study to derive population norms for the EQ-5D-5L by means of a postal survey based on a nationally representative sample of Norwegians adults. The norms are presented for the five dimensions, EQ-5D index, and EQ VAS scores across age (18–29, 30–39, 40–49, 50–59, 60–69, 70–79, ≥ 80 years), sex, and education level (below/above upper secondary school and short/long higher education).

## Methods

### Data collection

Age and sex-specific response rates to existing Norwegian postal surveys, including those designed to give population norms [11, 12, 27–29], were assessed and determined the necessary sample size to give a similar number of respondents, and estimates of equal precision, per age and sex group. The National Registry of the Norwegian Tax Administration (Folkeregisteret) was used to select a random group of 12,790 adults aged 18 years and over, who in December 2019, were sent a postal questionnaire and reply-paid envelope addressed to the Norwegian Institute of Public Health. The accompanying letter explained the purpose of the study, that by responding to the questionnaire they gave their informed consent, and that those returning a completed questionnaire would be included in a lottery of ten prizes each to the value of NOK 10,000 (1000 Euro).

The EQ-5D-5L was translated into the Norwegian language in accordance with EuroQol translation procedures including forward backwards translation, cognitive debriefing, and quality control [30]. Used alongside the EQ-5D, the EQ VAS is a 20 cm vertical visual analog scale assessing “your own health today” on a scale of 0 to 100 corresponding to the worst and best imaginable health. Responses to the five items representing EQ-5D-5L health states are identified by a five-digit number (for example, 11232), each of which corresponds to the response category reported for successive dimensions, beginning with mobility. Each state has a value attached, which are based on national value sets, typically derived from general population studies, and used in the calculation of QALYs. In the absence of a scoring algorithm for Norway, the Norwegian Medicines Agency recommends [8] use of the UK EQ-5D-3L value set [9] mapped to the EQ-5D-5L descriptions of health [10], a “crosswalk value set”. This crosswalk value set was used for the Norwegian EQ-5D-3L population norms [11] and is available to the system of national quality [31, 32]. The questionnaire also included questions relating to age, gender, and education level.

The Regional Committee for Medical and Research Ethics stated that the study did not require their approval. The Data Protection Impact Assessment was approved by the Norwegian Institute of Public Health on the 16th October 2019.

### Population norms

Presentation of norms including descriptive statistics, follow existing EQ-5D-5L studies and include EQ-5D-5L dimension, index, and EQ VAS scores for the entire sample and by seven age groups, sex and education level [2, 14–26]. The latter is less widely reported but has a consistent role in explaining lower EQ-5D scores [13]. The most widely reported health states are shown for EQ-5D-5L states with 0.5% or more responses. The data were weighted according to age, gender, and education level to improve representativeness of the Norwegian general population using data from Statistics Norway for 1 October, 2019. The latter were used to compute frequencies that adjust for over- or underrepresentation in relation to these characteristics.

Statistical analyses were performed in R version 4.0.4 [33].

## Results

### Data collection

Of the 12,790 questionnaires mailed, 426 were returned by the Post Office for being wrongly addressed, and one person had died. Of the remainder, 3200 (25.9%) returned a questionnaire that was at least partly completed. The results that follow are based on the 3120 (24.4%) respondents who completed the EQ-5D-5L dimensions and background questions. There were 73 fewer usable responses to the EQ VAS. The mean age (SD) was 50.9 (21.7) and ages ranged from 18 to 97 years (Table 1). There were approximately 10% more female respondents than male, and 239 to 691 respondents across seven age groups; the lowest number of respondents was for 80 years and above and the highest was for those 18–29 years of age. Respondents to the five education levels ranged from 282 to 862 for below secondary school to postgraduate higher education. Survey respondents are also over-represented for the youngest and oldest age groups, and highest education level. The final two columns of Table 1 show the number of respondents across these categories after weighting was applied.

**Table 1:** Respondent characteristics (*n* = 3120)

	Sample		Weighted sample^a^	
	*n*	%	*n*^b^	%
Female	1713	54.9	1555	49.9
Male	1407	45.1	1565	50.1
Age, years				
18–29	691	22.1	615	19.7
30–39	385	12.3	533	17.1
40–49	368	11.8	531	17.0
50–59	453	14.5	517	16.6
60–69	484	15.5	429	13.8
70–79	500	16.0	322	10.3
> = 80	239	7.7	174	5.6
Education				
Below upper secondary school	282	9.0	729	23.4
Upper secondary school	1215	38.9	1281	41.1
Higher education < 4 years	761	24.4	779	25.0
Higher education ≥ 4 years	862	27.6	330	10.6

^a^Using general population data from Statistics Norway (www.ssb.no/befolkning), 1 October, 2019

^b^Some columns do not sum to 3120 due to rounding errors

Table 2 shows that almost one-third of respondents reported no problems on any of the five EQ-5D-5L dimensions, and 23% reported slight problems in relation to self-care. Twenty health states had frequencies of 0.5% and above, accounting for 84.3% of respondents. Of the possible 3125 EQ-5D-5L health states, 277 (8.9%) were reported.

**Table 2:** Most frequently^a^ reported EQ-5D-5L health states

Health state	*n*	%	Health state	*n*	%
11111	1006	32.2	11113	30	1.0
11121	704	22.6	11132	30	1.0
11122	271	8.7	21122	26	0.8
11112	158	5.1	11223	24	0.8
21121	60	1.9	11231	22	0.7
11221	51	1.6	21222	20	0.6
11123	46	1.5	11122	16	0.5
11222	44	1.4	11112	16	0.5
21221	38	1.2	11232	16	0.5
11131	36	1.2	21231	15	0.5

^a^EQ-5D-5L health states reported by at least 0.5% of respondents

### Population norms

The remainder of the results relate to the weighted data. The distributions of the EQ-5D-5L item responses for the seven age groups are shown in Table 3 and separately for females and males in Tables 4 and 5, respectively. Irrespective of age and sex, the great majority (76–93%) reported no problems with mobility, self-care, and usual activities. Two exceptions are mobility and usual activities for those aged 80 years and over, where approximately 50% of females and 60% of males reported no problems (Tables 4 and 5). The great majority (83%) reported none or slight pain/discomfort. For anxiety/depression the majority (65%) reported no problems, which is more apparent for older age groups. Apart from anxiety/depression, the proportion reporting no problems decreases with age (Tables 3, 4 and 5).

**Table 3:** Weighted distribution^a^ of EQ-5D items scores by age (*n* = 3120^b^)

EQ-5D-5L item	Age group, years														All	
	18–29		30–39		40–49		50–59		60–69		70–79		> = 80			
	*n*	%	*n*	%	*n*	%	*n*	%	*n*	%	*n*	%	*n*	%	*n*	%
Mobility																
No problems	553	89.8	495	92.9	432	81.5	418	80.8	349	81.2	225	69.9	86	49.7	2558	82.0
Slight problems	39	6.4	26	4.9	68	12.9	63	12.3	53	12.3	65	20.3	44	25.3	359	11.5
Moderate problems	10	1.6	11	2.0	23	4.3	25	4.9	12	2.9	18	5.6	21	12.0	120	3.8
Severe problems	13	2.0	1	0.2	7	1.3	9	1.8	15	3.6	12	3.6	17	9.6	74	2.4
Unable to do	1	0.1	0	0.0	0	0.0	1	0.2	0	0.0	2	0.7	6	3.4	10	0.3
Self-care																
No problems	593	96.4	509	95.5	498	93.9	466	90.2	402	93.7	290	90.2	135	77.4	2893	92.7
Slight problems	14	2.2	16	2.9	12	2.2	36	6.9	20	4.7	22	6.8	23	13.1	142	4.5
Moderate problems	8	1.3	8	1.6	15	2.9	14	2.7	3	0.8	7	2.1	9	5.0	64	2.1
Severe problems	1	0.1	0	0.0	5	1.0	1	0.2	3	0.8	3	0.8	6	3.3	19	0.6
Unable to do	0	0.0	0	0.0	0	0.0	0	0.0	0	0.1	0	0.0	2	1.1	2	0.1
Usual activities																
No problems	470	76.4	454	85.2	378	71.3	372	72.1	345	80.3	250	77.8	94	54.2	2364	75.8
Slight problems	94	15.4	60	11.2	90	17.0	101	19.6	59	13.8	47	14.6	44	25.2	496	15.9
Moderate problems	27	4.3	15	2.7	46	8.7	27	5.2	15	3.4	9	2.7	19	11.2	157	5.0
Severe problems	17	2.8	3	0.5	13	2.4	16	3.1	11	2.5	13	4.1	8	4.8	81	2.6
Unable to do	7	1.2	1	0.2	4	0.7	0	0.0	0	0.0	3	0.8	8	4.6	23	0.7
Pain/discomfort																
None	302	49.0	243	45.6	166	31.3	181	34.9	150	35.0	97	30.3	44	25.0	1183	37.9
Slight	250	40.7	225	42.2	256	48.2	219	42.4	203	47.4	162	50.5	89	51.2	1404	45.0
Moderate	39	6.4	50	9.3	67	12.7	75	14.5	45	10.4	39	12.2	31	17.6	345	11.1
Severe	11	1.8	14	2.7	34	6.3	37	7.2	26	6.0	19	6.0	9	5.1	150	4.8
Extreme	13	2.1	1	0.2	7	1.4	5	1.0	5	1.2	4	1.1	2	1.1	37	1.2
Anxiety/Depression																
None	349	56.8	326	61.2	334	62.9	334	64.6	317	73.9	240	74.6	116	66.9	2016	64.6
Slight	143	23.3	134	25.1	121	22.7	130	25.1	78	18.2	66	20.6	45	25.8	716	23.0
Moderate	83	13.5	45	8.5	51	9.5	40	7.7	27	6.4	12	3.8	10	5.5	268	8.6
Severe	36	5.8	19	3.6	18	3.4	13	2.6	7	1.6	3	0.8	3	1.8	99	3.2
Extreme	4	0.6	9	1.6	7	1.4	0	0.0	0	0.0	1	0.2	0	0.0	21	0.7

^a^Using general population data from Statistics Norway (www.ssb.no/befolkning), 1 October, 2019. Frequency weights for age, sex, and education level

^b^Some columns do not sum to 3120 due to rounding errors

**Table 4:** Weighted distribution^a^ of EQ-5D items scores for females by age (*n* = 1555^b^)

EQ-5D-5L item	Age group. years														All	
	18–29		30–39		40–49		50–59		60–69		70–79		> = 80			
	*n*	%	*n*	%	*n*	%	*n*	%	*n*	%	*n*	%	*n*	%	*n*	%
Mobility																
No problems	263	88.1	243	93.2	210	81.3	195	77.3	172	80.2	108	65.2	48	45.3	1238	79.6
Slight problems	27	8.9	13	4.9	36	13.8	34	13.3	29	13.4	41	24.6	29	27.6	207	13.3
Moderate problems	9	2.9	4	1.4	11	4.2	15	5.8	5	2.4	10	6.2	15	14.0	68	4.4
Severe problems	0	0.1	1	0.5	2	0.7	8	3.2	9	4.1	5	3.2	12	11.1	37	2.4
Unable to do	0	0.0	0	0.0	0	0.0	1	0.4	0	0.0	1	0.8	2	2.0	5	0.3
Self-care																
No problems	285	95.5	253	97.3	243	94.2	219	86.7	204	95.4	155	93.6	79	75.1	1439	92.5
Slight problems	11	3.7	6	2.2	9	3.3	20	7.9	5	2.4	6	3.8	15	13.9	72	4.6
Moderate problems	2	0.6	1	0.5	6	2.5	12	4.9	3	1.3	3	1.9	8	7.4	36	2.3
Severe problems	1	0.2	0	0.0	0	0.0	1	0.4	2	0.9	1	0.8	4	3.6	9	0.6
Unable to do	0	0.0	0	0.0	0	0.0	0	0.0	0	0.0	0	0.0	0	0.0	0	0.0
Usual activities																
No problems	223	74.6	231	88.7	173	67.0	178	70.5	163	76.0	125	75.0	51	48.6	1143	73.5
Slight problems	56	18.8	22	8.6	50	19.3	44	17.6	39	18.1	30	17.9	30	28.4	271	17.4
Moderate problems	10	3.3	5	1.9	23	9.0	16	6.5	6	2.7	3	1.8	14	13.6	78	5.0
Severe problems	9	3.0	1	0.4	10	3.9	14	5.4	7	3.2	7	4.1	6	5.7	53	3.4
Unable to do	1	0.4	1	0.5	2	0.7	0	0.0	0	0.0	2	1.2	4	3.6	10	0.6
Pain/discomfort																
None	137	45.9	111	42.8	83	32.0	78	31.0	69	32.0	45	27.4	20	19.1	544	34.9
Slight	124	41.7	106	40.6	114	44.0	112	44.5	106	49.5	82	49.3	55	51.6	699	44.9
Moderate	25	8.4	39	14.9	41	15.9	29	11.6	23	10.7	26	15.8	22	20.7	205	13.2
Severe	10	3.4	3	1.2	19	7.3	28	11.1	13	5.9	12	6.9	8	7.4	92	5.9
Extreme	2	0.8	1	0.5	2	0.8	4	1.7	4	1.8	1	0.6	1	1.2	16	1.0
Anxiety/Depression																
None	158	52.9	162	62.4	159	61.6	157	62.2	154	71.9	118	70.8	65	61.4	973	62.5
Slight	83	27.8	74	28.5	74	28.5	62	24.6	41	18.9	41	24.4	31	29.1	405	26.0
Moderate	38	12.6	22	8.5	17	6.7	22	8.7	17	7.9	7	4.0	7	7.1	130	8.4
Severe	18	6.0	2	0.6	8	3.1	11	4.5	3	1.4	1	0.8	3	2.4	45	2.9
Extreme	2	0.7	0	0.0	0	0.2	0	0.0	0	0.0	0	0.0	0	0.0	3	0.2

^a^Using general population data from Statistics Norway (www.ssb.no/befolkning), 1 October, 2019. Frequency weights for age, sex, and education level

^b^Some columns do not sum to 1555 due to rounding errors

**Table 5:** Weighted distribution^a^ of EQ-5D items scores for males by age (*n* = 1565^b^)

EQ-5D-5L item	Age group. years														All	
	18–29		30–39		40–49		50–59		60–69		70–79		> = 80			
	*n*	%	*n*	%	*n*	%	*n*	%	*n*	%	*n*	%	*n*	%	*n*	%
Mobility																
No problems	290	91.5	252	92.6	222	81.6	223	84.2	177	82.3	117	75.0	39	56.5	1319	84.3
Slight problems	13	4.0	13	4.8	33	12.0	30	11.3	24	11.3	24	15.7	15	21.7	152	9.7
Moderate problems	1	0.3	7	2.6	12	4.4	11	4.0	7	3.4	8	4.8	6	9.0	51	3.3
Severe problems	12	3.9	0	0.0	5	2.0	1	0.4	7	3.1	6	4.0	5	7.3	37	2.3
Unable to do	1	0.3	0	0.0	0	0.0	0	0.0	0	0.0	1	0.5	4	5.5	5	0.3
Self-care																
No problems	308	97.2	256	93.9	255	93.6	247	93.5	198	91.9	135	86.7	55	81.1	1454	92.9
Slight problems	3	0.8	10	3.6	3	1.2	16	5.9	15	7.0	16	10.0	8	12.0	70	4.5
Moderate problems	6	1.9	7	2.6	9	3.2	1	0.6	1	0.3	4	2.4	1	1.2	29	1.8
Severe problems	0	0.0	0	0.0	5	2.0	0	0.0	1	0.6	1	0.9	2	2.9	10	0.6
Unable to do	0	0.0	0	0.0	0	0.0	0	0.0	0	0.2	0	0.0	2	2.8	2	0.1
Usual activities																
No problems	247	78.1	223	81.9	205	75.3	194	73.6	182	84.7	126	80.7	43	62.9	1221	78.0
Slight problems	38	12.1	38	13.8	40	14.8	57	21.5	20	9.5	17	11.0	14	20.1	224	14.3
Moderate problems	17	5.3	10	3.6	23	8.3	10	3.9	9	4.1	6	3.7	5	7.4	79	5.1
Severe problems	8	2.5	2	0.7	3	0.9	2	0.9	4	1.7	6	4.1	2	3.5	27	1.7
Unable to do	6	1.9	0	0.0	2	0.6	0	0.0	0	0.0	1	0.5	4	6.1	13	0.8
Pain/discomfort																
None	165	52.1	131	48.2	84	30.7	102	38.7	82	38.0	52	33.4	23	34.2	639	40.8
Slight	126	39.8	119	43.7	142	52.3	107	40.3	97	45.2	81	51.7	34	50.4	706	45.1
Moderate	14	4.5	11	3.9	26	9.6	46	17.3	22	10.1	13	8.3	9	12.8	140	9.0
Severe	1	0.3	11	4.2	15	5.5	9	3.4	13	6.1	8	5.0	1	1.6	58	3.7
Extreme	11	3.3	0	0.0	5	2.0	1	0.4	1	0.6	3	1.6	1	0.9	21	1.4
*Anxiety/Depression*																
None	191	60.4	164	60.1	175	64.2	177	67.0	163	75.9	122	78.6	51	75.3	1044	66.7
Slight	60	19.0	60	21.9	47	17.3	67	25.5	37	17.4	26	16.5	14	20.7	311	19.9
Moderate	45	14.3	23	8.5	33	12.2	18	6.8	11	4.9	5	3.5	2	3.1	138	8.8
Severe	18	5.7	18	6.4	10	3.8	2	0.8	4	1.8	1	0.9	1	0.9	54	3.4
Extreme	2	0.6	9	3.1	7	2.6	0	0.0	0	0.0	1	0.4	0	0.0	18	1.2

^a^Using general population data from Statistics Norway (www.ssb.no/befolkning), 1 October, 2019. Frequency weights for age, sex, and education level

^b^Some columns do not sum to 1565 due to rounding errors

Responses to the two categories of severe or extreme problems ranged from 0.7 to 6.0% for the self-care and pain/discomfort dimensions, respectively (Table 3). Responses to the two categories of severe or extreme problems for the mobility and self-care dimensions, came mostly from the two oldest age groups. Severe and extreme problems for usual activities, pain/discomfort, and anxiety/depression were more evenly distributed across age groups with two exceptions; the age group 30–39 years, which had among the highest levels of health for four dimensions, and the youngest age group, which had highest reported levels of anxiety/depression.

The EQ-5D index and EQ VAS scores are shown in Table 6. Compared to males, females have slightly lower scores for the EQ-5D-5L index and similar scores for the EQ VAS. For all respondents, the index and EQ VAS scores follow a similar pattern across the seven age groups, with slightly higher scores for the age group 30–39 years compared to the youngest age group. The scores fall slightly for the next age group 40–49 years, increase for the age groups 50–59 and 60–69, and decrease in the oldest age groups. These fluctuations are slightly higher for the EQ-5D index scores in males compared to females. Both scores show a slightly different pattern for females and males. Compared to the EQ-5D index scores for the age group 40–49 years, the female age group 50–59 years has an increase, while the same male group has a decrease in scores. This pattern is reversed for the age group 60–69 years. Compared to the EQ VAS scores for the age group 19–29 years, the female age group 30–39 years has a decrease, while the same male age group has an increase in scores.

**Table 6:** Weighted^a^ EQ-5D index and EQ VAS (0–100 scale) scores for the whole sample, age, gender, and education

	EQ-5D index			EQ VAS		
	*n*^b^	Mean	SD	*n*^b^	Mean	SD
All	3120	0.805	0.201	3120	77.9	18.3
Sex						
Female	1555	0.796	0.197	1555	78.0	18.8
Male	1565	0.814	0.204	1565	77.9	17.8
Ages						
18–29	615	0.820	0.198	615	78.9	16.1
30–39	533	0.839	0.177	533	80.8	15.8
40–49	531	0.788	0.210	531	75.0	20.5
50–59	517	0.799	0.202	517	78.3	17.9
60–69	429	0.816	0.184	429	80.2	17.7
70–79	322	0.793	0.199	322	78.0	18.1
≥ 80	174	0.716	0.254	174	67.8	23.9
Ages female						
18–29	317	0.825	0.211	317	79.4	15.7
30–39	272	0.828	0.208	272	78.7	16.6
40–49	272	0.786	0.223	272	74.8	20.3
50–59	264	0.831	0.161	264	79.5	15.4
60–69	215	0.826	0.177	215	79.7	18.1
70–79	156	0.805	0.209	156	77.6	18.4
≥ 80	68	0.746	0.270	68	68.6	22.4
Ages male						
18–29	298	0.814	0.184	298	78.4	16.4
30–39	260	0.850	0.136	260	82.9	14.6
40–49	258	0.790	0.196	258	75.2	20.7
50–59	253	0.767	0.233	253	77.0	20.0
60–69	214	0.806	0.190	214	80.7	17.4
70–79	166	0.781	0.189	166	78.4	17.8
≥ 80	106	0.697	0.243	106	67.3	24.9
Education						
Below upper secondary	729	0.724	0.255	729	71.9	21.8
Upper secondary	1281	0.810	0.188	1281	78.2	18.1
Higher education < 4 years	779	0.844	0.158	779	81.1	14.9
Higher education ≥ 4 years	330	0.874	0.142	330	82.8	14.1

^a^Using general population data from Statistics Norway (www.ssb.no/befolkning), 1 October, 2019. Frequency weights for age, sex, and education level

^b^Some columns do not sum to 3120 due to rounding errors

Both scores show a linear trend toward higher levels of health with the four levels of education from below upper secondary school level to postgraduate (Table 6). The differences between levels is very similar for the EQ-5D-5L and EQ VAS. The largest differences are for the first two levels of below and upper secondary school education. For both scores this difference is larger than that for the three remaining levels of education combined.

## Discussion

This study makes available the first Norwegian population norms for the EQ-5D-5L from the Norwegian general population. These data are highly important to Norwegian users of PROMs in clinical and health services research, and the Norwegian NQRs, where EQ-5D-5L is by far the most widely used patient-reported instrument. The survey was specifically designed for the collection of norm data, whereas published data for several countries followed the collection EQ-5D valuation data for national scoring algorithms [2, 14, 15, 17, 20] or as part of other health surveys [24, 25]. The former followed EuroQol valuation technology and survey requirements including computer-assisted face-to-face interviews and sample sizes of approximately 1000, although the EQ-5D-5L and EQ VAS are generally completed by means of pen and paper in this context [5, 14]. The presence of an interviewer may still contribute to social desirability bias [2].

Differences in survey design, methods of recruitment and reporting limit comparisons with EQ-5D-5L norms for those available from other countries. However, as was found for Norway, across the eleven countries for which EQ-5D dimension data were reported, older groups generally reported increasing problems apart from anxiety and depression [2, 14, 15, 17, 19, 20, 22–26]. There were some exceptions for the usual activities dimension in the age groups 60–79 years, which reported less problems than the younger age groups. Less health problems were found for age groups overlapping with 60–79 years for Hong Kong [17] and Ireland [20], but the differences were smaller and limited to the adjacent lower age group. Furthermore, compared to the youngest age group, those aged 30–39 years reported less problems with usual activities, which is comparable to the findings from five countries [15, 17, 19–21]. Higher levels of anxiety/depression in the youngest age groups were also found for the youngest age groups in four Asian countries and Slovenia [15, 17, 19, 21, 23].

Males generally reported less health problems than females across the EQ-5D-5L dimensions. For four of the seven age categories (30–39, 40–49, 60–69, 70–79), males reported more problems with self-care than females. Across the eight countries for which such data were reported, the findings were similar for two or three overlapping age groups for Bulgaria [14], China [15], Ireland [20] and Poland [22].

The use of national value sets for scoring the EQ-5D-5L limits the interpretation of EQ-5D index scores across countries. The additional use of a common value set, including the first EQ-5D-3L value set [9] with mapping [10], or summated rating scale based on the five items [20, 24], would aid interpretation but are rarely reported. EQ-5D index and EQ VAS scores did not consistently decrease with age, rather there was a slight increase for the second age group and two age groups from 50 to 69 years. Some increases in scores or leveling off with increases in age were found for ten countries reporting this data [2, 14–17, 19–21, 25, 26]. In common with all other countries apart from the USA [2], EQ-5D index scores were lower for females than males [14–17, 19–26]. The large proportion of respondents scoring at the ceiling for the EQ-5D index is another common finding [22]. However, the number of respondents reporting no problems across the five dimensions was at the lower end of the range of 35–61% for the USA and South Korea, respectively [22].

The use of postal administration followed published Norwegian surveys for collecting norm data for generic PROMs including the EQ-5D and SF-36, the most recent being reported in 2018 [11, 12, 27]. Independent of mode of administration, such surveys often have low response rates for older age groups, but the sampling methodology used here secured a relatively high number of respondents, which allowed the use of 10 years categories up to 80 years of age and over. Existing Norwegian surveys had smaller samples for older age groups [11, 12, 27], even when there has been a much larger sample [11, 12]. This makes the norm data more relevant for the interpretation of EQ-5D scores from Norwegian patients, who are often older than respondents to surveys designed to give general population norms [28, 29]. Except for Spain, where the data came from a much larger sample [24], existing national norm data for the EQ-5D-5L has not included an age group of 80 years or above.

The response rate of 26% was low, and a reminder might have increased response rates. However, reminders sent to over 9000 non-respondents to the first mailing would have made it costly. Based on published Norwegian surveys [11, 27], a low response rate was expected. The lottery incentive was included to mitigate this, albeit with what appears to be limited success. It is not possible to ascertain the impact of the lottery, but the most recent Norwegian postal survey designed to collect population norms for the SF-36, had a response rate of 20% before and 36% after one reminder [27]. One postal survey designed to collect Norwegian norms for the earlier version of the EQ-5D, which has three levels (EQ-5D-3L), used a sample frame based on the same Norwegian register for 2010. No reminders were used, but a lottery ticket for NOK 25 (2.5 Euros) was given to half the sample, and the overall response rate was 23% [11].

The present study adopted a similar approach to other countries based on quota sampling including age and sex [19, 23]. However, given the over-representation of females and those with higher education levels, the data were not fully representative of the Norwegian general population and were therefore weighted. The vast majority of other countries [2, 14, 15, 19–23, 25, 26], have not weighted their data to better approximate the characteristics of general population, and because the norms are often shown by age and sex, there is no need for the sample to have the same distribution of these variables as the general population [13]. While not all EQ-5D-5L studies included a comparison with the general population, those that have also found over-representation in relation to females [20], younger [21] and older age groups [20], and higher education levels [2, 14, 23, 25]. This may be problematic for the aggregated columns of all respondents, but users can easily weight the data to their own needs [34, 35]. The unweighted and weighted population norms are included in the supplementary files, and the data will be made available to Norwegian users, including the national quality registers.

### Study limitations

There is currently no Norwegian value set or scoring algorithm for the EQ-5D-5L. Norwegian data were being collected for this purpose [5], but was postponed because of the COVID-19 pandemic. Other countries have faced similar issues in the reporting of population norms [14, 26]. Norms for the EQ-5D index scores were based on the UK value set with a mapping algorithm, which follows current recommendations [8]. When a Norwegian value set becomes available, EQ-5D index norms will be updated accordingly, and users informed.

Electronic data collection, including internet administration, is increasingly used for collecting PROMs data, and this additional mode of administration would have strengthened the study. Instrument scores and measurement properties are generally comparable for electronic and pen and paper administration, but response rates tend to be lower, and respondents less representative for the former [36–38]. EQ-5D data from internet panels have been shown to give systematically different respondents and norms to those from pen and paper administration [2]. Response rates to Norwegian surveys that include PROMs and related instruments including those assessing patient experiences, give lower response rates for internet compared to postal surveys and particularly for older age groups [11, 39, 40]. Norwegian norm data for the earlier version of the EQ-5D with three levels were collected in 2010 by postal and electronic means, and while the authors concluded that methodological considerations limited the comparison of the two response rates, there were just 57 respondents over 71 years of age in the electronic survey compared to 175 in the postal survey [11]. National surveys of patient experiences of health services quality in Norway span two decades, and while there has been a steady increase in both the use and response rates to internet surveys, response rates to the postal component continue to be highest [39, 40].

## Conclusion

This study collected the first general population norms for the EQ-5D-5L and EQ VAS from a sample of the Norwegian general population by means of a postal survey. The data will improve the interpretation of EQ-5D scores in clinical and health services research and quality indicator use, including the National Quality Registers.

## Supplementary Information

Below is the link to the electronic supplementary material.

Supplementary file1 (XLSX 67 kb)

## Data Availability

The data will be available for download from the Norwegian Centre for Research Data (nsd.no).

## Abbreviations

NQR: National quality registers
PROM: Patient-reported health outcome measure
QALY: Quality adjusted life year
